# Attitudes Toward a Pre-authorized Concealed Opioid Taper: A Qualitative Analysis of Patient and Clinician Perspectives

**DOI:** 10.3389/fpsyt.2022.820357

**Published:** 2022-03-24

**Authors:** Theresa Bedford, Nkaku Kisaalita, Nathaniel R. Haycock, C. Daniel Mullins, Thelma Wright, Michele Curatolo, Lynette Hamlin, Luana Colloca

**Affiliations:** ^1^711 Human Performance Wing, En Route Care, Wright Patterson Air Force Base, OH, United States; ^2^Mental Health Service Line, Orlando Veterans Affairs Healthcare System, Orlando, FL, United States; ^3^Department of Pain and Translational Symptom Science, School of Nursing, University of Maryland, Baltimore, MD, United States; ^4^Department of Pharmaceutical Health Services Research, School of Pharmacy, University of Maryland, Baltimore, MD, United States; ^5^Department of Anesthesiology, School of Medicine, University of Maryland, Baltimore, MD, United States; ^6^Department of Anesthesiology & Pain Medicine, School of Medicine, University of Washington, Seattle, WA, United States; ^7^Uniformed Services University of the Health Sciences, Graduate School of Nursing, Bethesda, MD, United States; ^8^Center to Advance Chronic Pain Research, University of Maryland, Baltimore, MD, United States

**Keywords:** placebo effects, concealed (hidden) administration, opioid tapering, chronic pain, qualitative descriptive

## Abstract

Standard opioid tapers tend to be associated with increased patient anxiety and higher pain ratings. Pre-authorized concealed opioid reductions may minimize expectations such as fear of increased pain due to the reduction of opioids and, prolong analgesic benefits in experimental settings. We recently observed that patients and clinicians are open to concealed opioid tapering. However, little is known about the “why” behind their attitudes. Based on this lack of data, we analyzed qualitative responses to survey questions on patients' and clinicians' acceptance of a concealed opioid reduction for chronic pain. Seventy-four patients with a history of high dose opioid therapy and 49 clinicians completed a web-based questionnaire with open-ended questions examining responses to two hypothetical clinical trials comparing a concealed opioid reduction pre-authorized by patients vs. standard tapering. We used content analysis based on qualitative descriptive methodology to analyze comments from the patients and clinicians. Five themes were identified: informed consent; anxiety; safety; support; and ignorance is bliss, or not. These themes highlight the overall positive attitudes toward concealed opioid tapers. Our findings reinforce the importance of patient-centered care and are expected to inform the design of clinical trials from both the patient and clinician perspective. This qualitative study presents patients' and clinicians' attitudes toward hypothetical scenarios for a trial of pre-authorized reduction of opioids. The findings indicate positive attitudes and the relevance of engaging patients with effective decision-making processes.

## Introduction

In an effort to reduce the harms associated with long-term opioid use, the Department of Defense (DoD)/Veterans Affair (VA) and the Centers for Disease Control (CDC) have published guidelines that recommend limiting prescriptions of opioids for the management of chronic pain to no more than 90 morphine milligram equivalents (MME) a day (1–3). While there is burgeoning evidence regarding the potential benefits of opioid tapering (4–7), debate persists about tapering methods, challenges, and potential harms, including increased risk of drug overdoses and mental health crises (8–10). In addition, there is limited empirical evidence to support best practices for successful opioid tapering in patients with chronic pain (11, 12). We conducted a survey of both patients and clinicians to explore attitudes toward a pre-authorized concealed taper of opioids with the purpose of gathering critical knowledge to implement in future trials using a concealed taper (13). The research used a mixed-methods approach: closed-ended questions with forced-choice answers, followed by the opportunity for respondents to reply with open-ended comments. When presented with scenarios (13) related to a hypothetical trial with pre-authorized concealed taper of opioids, both clinicians and patients believed that a concealed taper is more likely to be successful than a standard taper. Nearly 50% of the patients who responded to the survey were willing to participate in the hypothetical trial of pre-authorized concealed taper of opioids, and almost 80% of clinicians were willing to refer patients to such a clinical trial. Patients and clinicians alike saw the proposed concealed opioid reduction as a possible way to mitigate clinical pain, opioid-related side effects, and withdrawal symptoms (13).

Analgesic benefits of opioids may be prolonged by minimizing negative expectations through the use of a concealed opioid reduction. Prior research has demonstrated that patient expectations directly influence pain outcomes. Higher pain ratings have been linked to fear of pain, increased stress, high anxiety, and pessimism, while lower pain ratings have been linked to reduced stress and anxiety (14). The simple act of telling a patient that their opioid dosages are going to be reduced elicits negative expectations, such as fear of increased pain and anxiety (15). Frank and colleagues (16) found that patients reported a fear of increased pain, opioid withdrawal, and insufficient non-opioid treatment options when they were told that their opioid dosage was going to be reduced.

While the empirical literature on blind opioid tapering is limited, our research group has written about how this novel approach may benefit patients with chronic pain (17). One experimental study found that patients rated their pain higher when told that morphine was going to be discontinued (18). However, when morphine discontinuation was concealed, patients did not report higher pain ratings, despite the decrease in medication. These results suggest that opioid doses may be most successfully reduced when the patient is unaware of the taper.

Transparency is an important construct in ethical research and concerns arise whenever when patients are misled (19–21). However, patients can be explicitly asked to agree to a concealed taper in which they will receive pills packs that intersperse full doses with reduced doses of opioid pain medication. While being informed that while their intake of opioids will gradually be reduced, they will not know exactly when the taper will occur. The patient's autonomy, the clinician's integrity, and societal trust in medicine are thereby preserved (21).

While hiding the interruption of opioids remains highly questionable in research and clinical practices (22, 23), patients can be pre-informed about the concealment of certain parts of the research (24), making the concealment ethically permissible (25–27) and, most importantly, agreeable to patients with chronic pain (28).

Research on attitudes toward blinded tapers suggests that patients with chronic pain and clinicians who manage patients with chronic pain may be open to a trial with a concealed opioid taper. In addition, studies examining clinicians' perspectives highlight several factors, such as effective communication, that may further bolster opioid tapering efforts (29–31). In this article, we performed content analyses of previously published survey responses to further examine perspectives toward pre-authorized concealed opioid taper scenarios. We have previously reported the quantitative findings of this study (13). The purpose of this qualitative analysis is to provide an in-depth understanding of attitudes toward a concealed taper strategy in both patients and clinicians. The results of this analysis will be used to guide the design of future approaches to concealed-taper designs that will optimize opioid tapering and chronic pain management.

## Methodology

This is a qualitative descriptive study based on written data from a cross-sectional survey. Quantitative findings from this cross-sectional survey examining patient and clinician acceptance of a concealed opioid reduction for chronic pain have been previously reported (13). Both patients and clinicians were recruited through advertisements on social media, online ads, flyers, and both local and non-local pain clinics from January 2018 to December 2019. As medical practice can vary by country, we chose to limit clinicians to those who practiced in the United States (U.S). We also chose to limit our patient sample to those who had taken or were currently taking at least 90 MME of an opioid. We wanted to capture the views of clinicians who were caring for patients on opioids such as registered nurses, family practice clinicians, anesthesiologists who manage pain, and others, as well as capture patients who could potentially participate in an opioid tapering clinical trial. A total of 74 patients who were currently taking or had taken high dose opioids (i.e., >90 MME) and 49 clinicians consented to participate. The survey was administered through REDCap, a secure HIPAA compliant survey and data management tool and took ~20 min to complete. This study was approved as non-human research by the University of Maryland, Baltimore Institutional Review Board (HP-00073609) and according to the definitions of the U.S. Department of Health and Human Services (HHS) and Food and Drug Administration (FDA).

Each patient respondent answered a 13-question survey including general questions related to demographic information and addressing their responses to two hypothetical patient scenarios of opioid-dose tapering. The first scenario depicted a standard tapering (overt administration) and the second scenario depicted a concealed dose tapering (covert administration). For details, see hypothetical scenarios below:

*Hypothetical scenario 1*.

“*A 46-year-old man had a back injury 10 years ago, for which he started taking 50 morphine equivalent daily dose (MEDD). Now, many years later, his dose has risen to 200 MEDD. His doctor wants to recruit him for a 6-week clinical study to help reduce his opioid use. The study consists of two groups: Group 1 (standard 6-week taper) receives a standard, gradual opioid taper (consistent with DoD/CDC recommendations) for 6 weeks. Throughout the study, the participant is monitored over the phone and provided supportive counseling and psychotherapy for chronic pain. Group 2 (concealed 3-month taper) is also told they will receive a gradual opioid taper for 3 months. However, during the informed consent process, the participant is informed that they will not be aware of how much their opioids are being decreased from week to week as the number of pills will remain the same. Throughout the study, the participant is monitored over the phone and provided supportive counseling and psychotherapy for chronic pain.”* [pg. 3, (13)].

*Hypothetical scenario 2*.

“*A 32-year-old woman is in the hospital for a few months after experiencing a terrible accident. She has been treated with morphine before and reports that it significantly helps decrease her pain. However, when she is administered morphine covertly (i.e., without her knowledge), her pain ratings significantly increase, even though she is receiving the same dose as when she is given morphine overtly (i.e., when she's aware of the administration). This shows that she is a placebo responder, since her knowledge of receiving morphine helps her feel less pain. She keeps asking her doctor to raise her dose of morphine. Her doctor is considering recruiting her for the same clinical study described in scenario 1 to reduce her dependence on opioids. Therefore, she will be told that she will not be aware of exactly when morphine will be given. The doctor believes she will have positive outcomes on this clinical study, which involves the placebo effect, since she is already known to be a placebo responder.”* [pg. 3, (13)].

Open-ended questions followed each closed-ended question (for details about the scenario-related questions, see Table 1), asking “Why or why not?” providing patients the opportunity to provide a rationale for their closed-ended answer to the hypothetical experiences in the two scenarios. There was one additional open-ended question that asked each patient “How do you feel about the opioid reduction recommendations?” Patients were not enrolled in a real-world clinical trial.

**Table 1:** Scenario-related questions.

**Patients' study questions**
Scenario 1, Do you think participants in Group 1 and 2 will have similar pain ratings and withdrawal symptoms?
Scenario 2, Do you think she is a good participant for the study?
Scenario 2, Do you think this study can help her smoothly wean off morphine if she is placed in Group 1?
Scenario 2, Do you think this study can help her smoothly wean off morphine if she is placed in Group 2?
Scenario 2, Since the woman from the scenario is known to be a placebo responder, do you feel that she has a better chance of responding positively to being in Group 2 over someone who is not known to be?
Do you think this study may help patients reduce the stress or anxiety that may be associated with reduction of opioids?
Would you feel comfortable participating in this study as a patient?
Overall, do you think it is important to reduce the amount of opioids prescribed to patients in the US today?
**Clinicians' study questions**
Scenario 1, Do you think participants in Group 1 and 2 will have similar pain ratings and withdrawal symptoms?
Scenario 2, Do you think she is a good participant for the study?
Scenario 2, Do you think this study can help her smoothly wean off morphine if she is placed in Group 1?
Scenario 2, Do you think this study can help her smoothly wean off morphine if she is placed in Group 2?
Scenario 2, Since the woman from the scenario is known to be a placebo responder, do you feel that she has a better chance of responding positively to being in Group 2 over someone who is not known to be?
Do you think this study may help smoothly wean heavy opioid users down to a lower dose?
Would you feel comfortable referring patients for this study?
Overall, do you think it is important to reduce the amount of opioids prescribed to patients in the US today?

Each clinician respondent answered a clinician-specific 11-question survey on the same two hypothetical scenarios of opioid-dose tapering, including general questions related to demographic information and area of specialty. Nine of the questions contained close-ended responses, followed by an open-ended response “Why or why not?” The remaining open-ended question asked each clinician “Do you think that this recommendation is justified? Please elaborate on your response.” The clinician questionnaire differed from the patient questionnaire in that it focused on the clinicians' responses to the patients' hypothetical participation in the two scenarios.

### Data Analysis

Data were analyzed using content analysis, a method applicable to both quantitative and qualitative approaches (32). Krippendorff (33) defined content analysis as a “research technique for making replicable and valid inferences from texts to the context of their use” [p. 18 (33)]. For the purpose of this analysis, content analysis was used to determine the presence of themes within the written responses of the patients and clinicians. Rather than interviewing patients with open-ended questions, we sought to understand their responses to our quantitative survey through their written word. While content analysis can use either a deductive or inductive approach, we chose an inductive approach. Inductive content analysis involves the use of abstraction and the formation of concepts or themes in order to reduce data, group it, and ultimately to answer the study questions (34). The inductive approach is recommended when little is known about the phenomenon being studied (35).

Emergent coding was used to establish codes after an examination of the data (36). Two of the authors (coders) independently read and examined the open-ended responses to search for themes that captured patients' and clinicians' attitudes toward the new opioid guidelines and the two hypothetical scenarios. Then, the two coders used an inductive coding system as there is little, if anything, known about this phenomenon. Once each author had coded the data, they came together to discuss their individual results and obtained consensus. In content analysis, reliability is measured by stability and reproducibility (36). Stability refers to intra-rater reliability. Each of the two coders achieved the same results 95% of the time. Reproducibility refers to inter-rater reliability. Each of the two coders classified the data the same way at a rate of 90%.

SPSS version 22 was used for the analyses of demographic characteristics and frequency of themes.

## Results

We surveyed 74 patients and 52 clinicians. Three clinician surveys were removed because they practiced in a country outside of the United States. A total of 39 responses from patients and 64 comments from clinicians were reviewed. Some respondents, both patients and clinicians, provided several responses to individual open-ended questions, while some respondents provided no responses to the open-ended questions. The overall response rate to the open-ended questions for clinicians was 69% (*n* = 36) and for patients was 42% (*n* = 31).

The mean age of patients was 45 years (SD 12.608). Most patients identified as Caucasian (55.4%) and were currently using opioids (85.1%). Most clinicians identified as Caucasian (71.4%) and female (57.1%), with a mean age of 40 years (SD = 13.670). The largest group of clinicians were anesthesiologists (28.6%), followed by registered nurses (22.4%). This data has previously been reported in Bedford et al. (13).

The vocabulary density of the clinician survey was 0.332 and the vocabulary density of the patient survey was 0.343. Vocabulary density is the ratio of the number of words in the document to the number of unique words in the document. The lower vocabulary density results indicate that each survey's open-ended responses contained dense text with many single-use words.

The qualitative analysis resulted in the identification of five interrelated themes: (1) informed consent, (2) anxiety, (3) safety, (4) support, and (5) ignorance is bliss, or not. The two coders did not have any preconceived definitions of these themes. The two coders grouped each individual response and then re-examined those groupings of responses, moving from specific responses to the general themes identified (35). Table 2 presents the frequency of responses by clinicians and patients within each theme.

**Table 2:** Frequency of themes.

**Theme**	**Patient responses**	**Clinician responses**
Informed Consent	*N* = 9	*N* = 13
Anxiety	*N* = 5	*N* = 14
Safety	*N* = 6	*N* = 11
Support	*N* = 12	*N* = 13
Ignorance is bliss, or not	*N* = 7	*N* = 13
Total responses	39	64

### Informed Consent

Both patients and clinicians emphasized the importance of informed consent in a clinical trial. Responses grouped in this theme included, but were not limited to, words referencing informed, informed consent, procedures, monitoring, and choice. Clinicians stressed the need to “fully inform” patients of the dosing regimens. Clinicians underscored the need to provide patients with “knowledge” about the plan because the degree of knowledge which patients have may influence their pain. One clinician wrote: “Her pain is proportional to her mental state and knowledge of the administration of drugs” while another clinician highlighted the importance of informing the patients about randomization: “As long as [a] patient is fully informed of the potential for randomization to two different groups, then it is patient's choice whether or not to join study.”

Patients wanted to ensure that the benefits and risks were openly discussed with them. Patients emphasized the need to have knowledge about the tapering process and understanding the procedures. One patient wrote that “Knowing what is happening and having a planned reduction is much better than what many people are going through, which is a sudden reduction without any counseling on how to deal with the withdrawal symptoms.” Another patient suggested that informed consent could motivate patients. Another patient wrote “To adequately explain the benefits and all the data to support decreased opioid use. Also, the long-term detriment of opioids would help motivate someone to lower their dosage.”

One patient highlighted the negative consequences associated with not providing a thorough informed consent. The patient wrote, “people who are living without the correct level or amount of control, knowledge, and education on a subject, or thing... often are hostile when presented with any kind of authority […] they feel small and [resort] to the [‘] FIGHT OR FLIGHT[‘] thing in the brain.”

### Anxiety

For this theme, patients and clinicians described anxiety around reducing opioid dosages in open and concealed settings. Some clinicians were hesitant to enroll patients in the hypothetical clinical trial due to the concealed taper, such as this clinician: “I like the theory behind group 1 however group 2 I am hesitant about because I do not like how the participant doesn't know how much of their medications is decreased.” However, other clinicians thought the study design would be helpful for patients whose pain outcomes were related to knowing when they were receiving a pain medication: “Not knowing doses takes a lot of the focus off of that issue and reduces anxiety/nocebo. I think Group 2 would do better.”

Both patients and clinicians were supportive of the concealed opioid reduction trial with a focus on gradual reduction. One patient wrote: “I think the study would be good because you have to taper slowly so they don't have the ache and withdrawals of wanting it more. Being on a tapering schedule with monitoring would be helpful.”

However, both patients and clinicians had mixed views on whether the patient presented in the hypothetical scenarios would benefit from Group 2 (a concealed taper). Many patients described anxiety associated with pain and reducing the dose of opioids they were taking. Patients and clinicians believed that there was a “mental” component to the hypothetical patient's pain that would influence her outcomes. Some patients and clinicians did not believe the hypothetical patient would benefit because “not knowing” would create anxiety for her, while other patients and clinicians believed that “not knowing” would help. For example, one patient believed the hypothetical patient would not benefit because “if her mind knows that amount of dose is reduced she starts feeling pain therefore she should be placed in group 2 where she will not know about how much amount of dose is reducing” and a clinician wrote “I believe their withdrawal symptoms will be the same but their pain ratings will differ between group 1 and group 2. Notably, Group 2 will rate higher just because they do not know how much their opioids were decreased.”

### Safety

Many patients and clinicians supported tapering opioids for safety reasons. Respondents centered on this theme spoke about the dangers of opioids. Both patients and clinicians suggested that opioids were over-prescribed, difficult to taper, and risky to take long term. Clinicians wrote about a lack of evidence to support opioid use and the need to prescribe opioids judiciously. One clinician wrote: “In the U.S., we consume inordinate amounts of opioid medications. We need to set expectations appropriately. Opioid addiction and overdoses have very grave consequences.”

Patients focused on the need for a slow opioid taper and to minimize withdrawal symptoms. One respondent found the DoD/CDC guidelines (2) to be justified for two specific reasons: “(1) Addiction. (2) Misuse and abuse. You'll have patients take their medication home and then sell or give it to other people or whatever it is they do that doesn't use it for its intended purpose. (3) I think it's being given out like candy and we're winding up seeing people actually die and they're not dying from whatever their initial problem is but they're dying from the opioids.”

### Support

Both patients and clinicians highlighted the importance of providing the patient with support throughout the tapering process. They wrote about the hypothetical study design and specifically liked the use of monitoring, counseling, and education in both groups in the study, all reflective of the need for support during the hypothetically proposed study. One patient wrote: “It makes a big difference when you feel like you're not doing it alone.”

Another patient thought the study design would provide the support the patient needed for a successful taper: “Every time [the hypothetical patient in the scenario] sees the doctor, she is fine. So, if she was in group 2, and getting lower doses and not knowing it but still seeing the doctor, I believe that she would be fine and it would work better for her.” Clinicians had varying views on the amount and types of support that would be beneficial for the hypothetical patient. For example, “Beyond being a placebo responder … she did not also have much response to opioids.” The standard “supportive counseling and psychotherapy for chronic pain” may not be adequate and “I think a supported wean with no ‘set goal’ is probably one of the few ways to do this right.”

### Ignorance Is Bliss, or Not

Many patients and clinicians highlighted the psychological factors that influence pain. Responses that were grouped in this theme expressed two opposing beliefs: that not knowing one's dosages would be a positive attribute of this study, or (the opposite) that not knowing the dosage would have a detrimental psychological effect on the patient's pain levels. Some wrote about the psychological benefits of not knowing if they were receiving a concealed reduction. Many patients viewed the hypothetical patient presented in the concealed dosing scenario as being the best fit for the concealed reduction trial. “Out of sight out of mind” and “Yes because she starts feeling pain if she knows her dose is reduced therefore group 2 is suitable for her where she will not know about amount of dose reduced with time” are two examples. However, other patients did not think either group would be helpful because the hypothetical patient's pain was directly related to her “knowing” if she is receiving the medication: “I don't think she would make a good participant because the clinician will not know if her pain is real or not.”

On the other hand, some clinicians thought that the concealed reduction group would not help the hypothetical patient. One clinician wrote that “not knowing could push her to exaggerate her pain/ withdrawal symptoms at all times.” Another clinician wrote that “just knowing they're tapering, will cause opioid users' pain.”

Some patients personally recognized the psychological aspects of tapering. One patient wrote “If not knowing my dose helps me out mentally in terms of the calmness level and [I] have less side effects physically. The mental always goes from the mental to the physical. It'd be weaning me off.” Another patient wrote “Most of it isn't mental but yes there is a mental part to pain, and there's a medical part to knowing you're being cared of by a physician. Basically you're trying to trick people into not knowing their dose is lower since their pain is the same. Which is why I would be a willing participant even though I wouldn't want to.”

## Discussion

In this study, we explored patient and clinician openness toward a concealed reduction of opioids using qualitative data from a web-based survey. We analyzed 39 qualitative comments for common themes from patients and 64 comments from clinicians. Comparing patient and clinician perspectives toward a pre-authorized concealed reduction, five themes emerged: (1) informed consent, (2) anxiety, (3) safety, (4) support, and (5) ignorance is bliss, or not.

Our findings expand upon the work done by James et al. (37) and our previous quantitative study results (13), which found overall positive patient and clinician attitudes toward a clinical trial with a concealed reduction of opioids. That study showed that nearly 60% of patients were comfortable participating in the hypothetical study and 80% of clinicians were willing to refer patients to the hypothetical study. The high rate of willingness for most patients and clinicians in that study to participate or refer patients to participate, respectively, suggests that a pre-authorized concealed reduction is viewed as a viable alternative to standard opioid tapers. This noteworthy positive response is likely to be related to the pre-authorization approaches (20, 38–40) whereby patients and clinicians agree in concealing the time when opioids are weaned in order to enhance positive bodily responses (i.e., placebo effects) while minimizing negative one (i.e., nocebo effects, anxiety) (27). Both patients and clinicians described specific psychological benefits of a concealed taper and its influence on pain responses. This suggests that knowledge about the benefits of a concealed taper may be used to create positive expectations, which may in term minimize negative expectations and improve the success of a pre-authorized concealed taper (20, 38–40).

Themes of negative affect, safety, support, and tapering ambivalence have been identified in other qualitative studies of opioid tapering. Frank et al. (16) showed how patients reported experiencing fear of worsening pain and withdrawal symptom from tapering and reported feeling uncertain about the effectiveness of non-opioid treatments. The authors showed how patients tended to be less focused on the long-term risks of chronic opioid use, including overdoses. Patients in their study also identified factors associated with successful tapering, including safety (e.g., trusting their clinician; similar pain with fewer side effects) and support (e.g., access to social support resources) (16). Concerns regarding the perceived limitations of alternative pain control methods have also been noted in other studies (41).

Studies of clinicians' perspectives on opioid tapering have emphasized the importance of effective communication and patient-centered care (29, 31). One study found that the most common reason patients were hesitant to participate in a double-blinded randomized control trial for opioid tapering was lack of information (37). For patients, opioid tapering can be a dynamic experience that changes daily due to various medical and psychosocial factors that are often not fully communicated to clinicians, which further highlights the importance of open communication (31). Matthias et al.'s (29) study in patients and clinicians highlighted the benefits of individualized tapers, understanding the patients' perspectives, promoting an environment of support (e.g., ensuring patients will not feel abandoned), and communicating tapering benefits (29). A focus group study of primary care clinicians also noted the importance of empathizing with patients, utilizing individualized tapers, and having access to resources to support a patient-centered tapering approach (30).

Our study found that patients and clinicians were most open to a pre-authorized concealed reduction in the setting of informed consent. Patients emphasized their need to understand the benefits, while clinicians were comfortable referring patients for the hypothetical clinical trial when patients were fully informed about both groups (standard taper vs. concealed taper). Both patients and clinicians recognized the challenge in providing informed consent when concealment is part of the study design. We recommend consenting patients at the beginning of the study using the patient-centered “authorized” concealed opioid taper or standard taper. Patients would need to consent to enrollment into either group in order to participate in the study. Those assigned to the patient-centered “authorized” concealed opioid taper should be willing to accept that they may not know the time or dose of the opioid they would receive. Preliminary research suggests that some patients with chronic pain are generally open to the use of authorized deception in research (28). As pointed out by our study respondents, informed consent may help patients recognize the benefits of a pre-authorized concealed taper and/or develop a plan to manage withdrawal symptoms, thereby optimizing the patient-clinician communication and alliance.

Despite the benefits of informed consent, we found that many patients reported anxiety about reducing their opioid dosage blindly. However, our study was unique in that it also found that clinicians, too, were concerned about patients experiencing anxiety with a concealed taper. Researchers need to consider how the patient will be feeling at each stage of the taper based on the speed and dosage reduction of the taper (42). Communication about the benefits of a concealed taper may increase patients' comfort about enrolling in the study and clinicians' willingness to refer patients. Patient concerns should be addressed at the beginning of the taper and throughout the clinical trial. Researchers are encouraged to support patients by regularly assessing for anxiety, by fostering strong patient-clinician relationships with open communication, and by prescribing medications for symptomatic management of withdraw as indicated (16). In addition, use of multimedia, such as narrative videos, has been shown to bolster patient tapering self-efficacy and effectiveness and could be used during study enrollment (43). These resources could potentially further decrease anxiety and promote tapering acceptance if they highlight the expectancy-based mechanisms underlying the efficacy of concealed tapering (44, 45).

Both patients and clinicians in our study reported that some patients may not be candidates for the hypothetical clinical trial that we described. Researchers have an obligation to minimize patient harm by developing specific inclusion and exclusion criteria. Researchers may want to consider exclusion criteria for patients who think concealment will increase their pain, whereby only patients who believe their pain will stay the same or be reduced with the clinical trial should be permitted to enroll. Alternatively, given that prior therapeutic experience rather than expectations can trigger placebo effects (46), those who consent can still be enrolled knowing that conditioning (e.g., exposure to full doses of opioids and reduced doses), along with education, can still result in effectiveness of the taper, despite the negative expectations. Additionally, for patients with comorbid diagnoses of opioid use disorder or complex opioid dependence, it may be more appropriate to use other well-established, evidenced-based treatments for opioid-use disorder, such as buprenorphine (47).

Our findings highlight the importance of integrating the diverse perspectives of patients and other relevant stakeholders (e.g., caregivers, clinicians, researchers) to successfully translate these results into applied experimental and clinical settings (17). The Patient-Centered Outcome Research Institute (PCORI) published a 10-step patient engagement framework which would be instrumental in guiding the next steps in this line of research (48–50). Specifically, the 10-step framework is a model that can be used to integrate census opioid tapering recommendations with novel concealed opioid tapering approaches (51). Core patient engagement principles include shared decision making (e.g., involving patients in decisions regarding study design/implementation), co-learning (e.g., stakeholder participation on data safely monitoring boards), and partnership (e.g., patient engagement in dissemination of research results).

### Strengths and Limitations

The major strength of this study is the rich perspectives that both patients and clinicians shared in response to the two hypothetical scenarios. Content analysis is an unobtrusive method to directly analyze communication as text. These results, in addition to previously published quantitative data (13), provide significant insight and support for the best research methodology to implement when studying opioid tapering.

Limitations in our study stemmed from the qualitative design and the study population. Content analysis, by its very nature, involves some level of subjective interpretation. Findings from this study were limited to the responses that respondents provided. Some respondents chose to answer more of the open-ended responses than others. We do not know why some respondents did not answer each open-ended question. We do not know if respondents may have answered the open-ended questions differently if the questions were asked in-person. The use of other qualitative methods/designs (e.g., focus groups, in-depth interviews) may have provided greater context for these results. Finally, it is noted that this study represented responses to hypothetical vignettes. While patients and clinicians were asked to respond as if they were participating in or referring patients to the hypothetical clinical trials, it is possible that their actual responses may be different if they were participating in real clinical scenarios. Despite these limitations, the findings from this study are important and serve as a baseline for future research and study design in the area of concealed opioid reduction.

## Conclusion

Our study provides patient and clinician perspectives for a concealed opioid taper clinical trial. We identified five common themes among patients and clinicians to describe their attitudes toward concealment: (1) informed consent, (2) anxiety, (3) safety, (4) support, and (5) ignorance is bliss, or not. Our study emphasizes the need to consider patients' and clinicians' perspectives when designing clinical trials to support a patient-centered approach and improve both clinical applicability and patient outcomes. Our study supports the development of clinical trials with strong informed consent processes that improve patient anxiety and minimize harm, optimize patient support, and mitigate the psychological factors that exacerbate pain during opioid tapering.

## Data Availability Statement

The raw data supporting the conclusions of this article will be made available by the authors, without undue reservation.

## Ethics Statement

The studies involving human participants were reviewed and approved by University of Maryland Baltimore. Written informed consent for participation was not required for this study in accordance with the national legislation and the institutional requirements.

## Author Contributions

TB conducted the study, data analyses, and drafted the manuscript. NK helped with drafting the manuscript. NH conducted the study, analyses, and commented on the final draft. CM, TW, and MC commented on the final draft. LH helped supervise the data analyses, interpreting the results, and writing the manuscript. LC designed the study, supervised the team, and wrote the manuscript approving the final version. All authors contributed to the article and approved the submitted version.

## Funding

This work was funded by the Agency for Healthcare Research and Quality (R24HS022135, LC), MPowering the State Initiatives: Strategic Partnership Grant (LC), and University of Maryland Center for Addiction Research, Education, and Service (CARES, LC).

## Author Disclaimer

The opinions expressed herein are those of the authors and are not necessarily representative of the official policy or position of the Uniformed Services University of the Health Sciences, Department of Defense, Department of Health Affairs, or United States Air Force. The views expressed here are the authors own and do not reflect the position or policy of the Agency for Healthcare Research and Quality, Maryland State and University of Maryland or any other part of the federal and state government.

## Conflict of Interest

The authors declare that the research was conducted in the absence of any commercial or financial relationships that could be construed as a potential conflict of interest.

## Publisher's Note

All claims expressed in this article are solely those of the authors and do not necessarily represent those of their affiliated organizations, or those of the publisher, the editors and the reviewers. Any product that may be evaluated in this article, or claim that may be made by its manufacturer, is not guaranteed or endorsed by the publisher.
